# Prostaglandin E2 Reverses Aberrant Production of an Inflammatory Chemokine by Microglia from Sandhoff Disease Model Mice through the cAMP-PKA Pathway

**DOI:** 10.1371/journal.pone.0016269

**Published:** 2011-01-27

**Authors:** Eri Kawashita, Daisuke Tsuji, Masahiro Toyoshima, Yosuke Kanno, Hiroyuki Matsuno, Kohji Itoh

**Affiliations:** 1 Department of Medicinal Biotechnology, Institute for Medicinal Research, Graduate School of Pharmaceutical Sciences, The University of Tokushima, Tokushima, Japan; 2 Department of Clinical Pathological Biochemistry, Faculty of Pharmaceutical Science, Doshisha Women's Collage of Liberal Arts, Kyoto, Japan; 3 National Institute of Biomedical Innovation (NIBIO), Osaka, Japan; Brigham and Women's Hospital, Harvard Medical School, United States of America

## Abstract

**Background:**

Sandhoff disease (SD) is a neurodegenerative lysosomal β-hexosaminidase (Hex) deficiency involving excessive accumulation of undegraded substrates, including terminal GlcNAc-oligosaccharides and GM2 ganglioside. Microglia-mediated neuroinflammation contributes to the pathogenesis and progression of SD. Our previous study demonstrated that MIP-1α, a putative pathogenic factor for SD, is up-regulated in microglial cells derived from SD model mice (SD-Mg) through activation of Akt and JNK.

**Methodology/Principal Findings:**

In this study, we first demonstrated that prostaglandin E2 (PGE2), which is one of the lipid mediators derived from arachidonic acid and is known to suppress activation of microglia, reduced the aberrant MIP-1α production by SD-Mg to the same level as by WT-Mg. PGE2 also attenuated the activation of Akt and JNK. The inhibition of MIP-1α production and the activation of Akt and JNK occurred through the EP2 and 4/cAMP/PKA signaling pathway in the murine microglia derived from SD model mice.

**Conclusions/Significance:**

We propose that PGE2 plays a role as a negative regulator of MIP-1α production in the pathogenesis of SD, and that PGE2-EP2 and 4/cAMP/PKA signaling could be a target pathway for therapy for SD.

## Introduction

Sandhoff disease (SD) is an inherited lysosomal storage disease caused by a defect of the β-hexosaminidase (Hex) β-subunit gene (*HEXB*) associated with deficiencies of HexA (αβ) and HexB (ββ) [1], [2]. In SD patients, excessive accumulation of undegraded substrates including GM2 ganglioside (GM2) with a terminal *N*-acetylgalactosamine residue and oligosaccharides with a terminal β-linked *N*-acetylglucosamine residue (GlcNAc-oligosaccharides) is observed, particularly in neurons, due to the deficiencies of HexA and HexB, which leads to neurological symptoms in the central nervous system (CNS), such as the startle response, mental retardation, spasms and quadriplegia.

Microglia-mediated inflammation is involved in the pathogenic mechanisms underlying several neurodegenerative diseases, including Alzheimer's disease, human immunodeficiency virus (HIV)-associated dementia and Parkinson's disease [3], [4]. In the brains of Sandhoff disease model mice (SD mice) at the symptomatic stage [5], microglial activation is also a cause of neuroinflammation in the CNS of the mice [6]–[9]. Our earlier studies demonstrated that macrophage inflammatory protein-1α (MIP-1α is up-regulated selectively in the brains of SD mice during the pathogenesis, and in microglial cells derived from SD mice (SD-Mg) [10], [11]. Wu and Proia also demonstrated that MIP-1α is responsible for recruitment of macrophage/microglia from the periphery in the pathogenic process of SD, and that deletion of the MIP-1α gene increases the life span of SD mice [12]. These studies suggest that MIP-1α should be one of the putative pathogenic factors for SD, and down-regulation of the abnormal production of MIP-1α in the brain should delay the onset or progression of SD. However, no therapeutic approach to reduce the production of MIP-1α in the brain of SD has been reported.

PGE2 is one of the lipid mediators derived from arachidonic acid. PGE2 is widely known as an inflammatory mediator; a pivotal mediator in the induction of inflammation and anti-inflammation [13], [14]. The complexity of the function of PGE2 depends on the PGE2 receptor subtype, *i.e.* EP1, EP2, EP3 or EP4, due to their distinct and antagonistic signaling cascades. The EP2 and EP4 receptors couple to G_s_ to increase intracellular cAMP, whereas EP3 couples to G_i_ to decrease the cAMP level; EP1 couples to G_q_ to activate phospholipase C and increase the intracellular calcium concentration [15]. Several studies indicated that the PGE2-EP2 signaling cascade mediates neuroinflammatory response in neurodegenerative models [16], [17]. Interestingly, the PGE2-EP2 receptor is also involved in preventing LPS-induced inflammation in microglia [18], and has neuroprotective effects on glutaminate toxicity and cerebral ischemia [19], [20].

Recent studies on the periphery indicated that PGE2 inhibits the production of MIP-1α by dendritic cells and macrophages stimulated with LPS [21], [22]. However, the inhibitory effects and the underlying mechanism in the CNS are poorly understood. In this study, we examined whether PGE2 could suppress the MIP-1α production in SD-Mg and possess the therapeutic potential or not. We demonstrated for the first time that PGE2 can suppress the production by attenuating the activation of Akt and JNK through the EP2 and 4/cAMP/PKA pathway in SD-Mg.

## Materials and Methods

The animal experiments in this study were approved by the Animal Research Committee of the University of Tokushima (Approval ID: Tokudoubutsu10106).

### Materials

PGE2, Butaprost, PGE1 alcohol, Sulprostone, AH6809 and GW627368X were purchased from Cayman Chemicals (Ann Arbor, MI). Forskolin and adenosine 3′, 5′-cyclic monophosphate, N6-benzoyl sodium salt (6-Bnz-cAMP) were from Calbiochem Corp. (La Jolla, CA). 8-(4-Chlorophenylthio)-2′-O-methyladenosine 3′, 5′-cyclic monophosphate monosodium hydrate (8-pCPT-2′O-Me-cAMP) was purchased from Sigma Chemical Co. (St. Louis, MO.).

The mouse anti-phosphorylated Akt (Ser473) antibody, rabbit anti-phosphorylated SAPK/JNK, and rabbit anti-Akt and anti-SAPK/JNK antibodies were obtained from Cell Signaling Technology (Beverly, MA). The horseradish peroxidase (HRP)-labeled anti-mouse and HRP-labeled anti-rabbit IgG antibodies were from Amersham Pharmacia Biotech (Uppsala, Sweden) and GE Healthcare Bio-Sciences (Little Chalfont, UK), respectively.

### Cell culture

Microglial cell lines were prepared from the cerebra of 1-day-old SD (*Hexb^–/–^*) [5] and WT (*Hexb^+/+^*) mice as described previously [11]. Briefly, the cerebra were passed through a 300 µm nylon mesh in Hank's balanced salt solution (HBSS) using a cell scraper (Sumitomo Bakelite Medical, Tokyo, Japan), and then the cell suspension was plated on ϕ100 mm dishes. The mixed glial culture, including astrocytes and microglia, was maintained in Dulbecco's modified Eagle's medium (DMEM), supplemented with 10% fetal bovine serum (FBS), 5 µg/mL insulin and antibiotics, under 5% CO_2_ at 37°C for about 2 weeks. Then the mixed glial culture was maintained in presence of 1 µg/mL granulocyte-macrophage colony-stimulating factor (GM-CSF) for a certain period. The floating GM-CSF-responsive microglial cells were collected and maintained in DMEM containing 10% FBS, 5 µg/mL insulin, 1 µg/mL GM-CSF and antibiotics, to establish the microglial cell lines. The obtained cell lines were maintained in DMEM supplemented with 10% FBS, 5 µg/mL insulin, 1 µg/mL GM-CSF and antibiotics.

### Enzyme-linked immunosorbent assay for MIP-1α production

SD-Mg plated on 96-well plates (2×10^4^ cells/well) were treated with reagents including PGE2, PGE2 analogs and cAMP analogs for 6 h. The conditioned medium (CM) was centrifuged at 2,300×g for 5 min. The MIP-1α levels in aliquots of the CM were measured with a mouse MIP-1α immunoassay kit (DY450; R&D Systems, Minneapolis, MN). In this assay, cell viability was also evaluated by means of the Tetra Color One cell proliferation assay system (Seikagaku Kogyo,Tokyo, Japan).

### Reverse transcription–polymerase chain reaction (RT-PCR) analysis

Total RNA was isolated from SD- and WT-Mg (each 5×10^5^ cells/dish) using TRIsure (Bioline, London, UK), and 1 µg of RNA from each sample was transcribed using ReverTra Ace-α- (TOYOBO, Osaka, Japan) according to the manufacturer's protocol. RT products were diluted three-fold. PCR for murine MIP-1α and glyceraldehyde 3-phosphate dehydrogenase (GAPDH) mRNAs was performed as follows: MIP-1α and GAPDH mRNAs were amplified in reaction mixtures consisting of 5 µL of Go Taq reaction buffer (Promega, Madison, WI), 0.5 µL of 10 mM dNTPs, 0.625 U of Go Taq DNA polymerase and 0.5 µM of each primer. The primer sets used for MIP-1α were described previously [11]. The primer sequences for GAPDH were 5′-TTCATTGACCTCAACTACATG-3′ (forward) and 5′-GTGGCAGTGATGGCATGGAC-3′ (reverse). The PCR conditions were as follows: 5 min at 94°C for denaturation, then 30–33 cycles of denaturation at 94°C for 30 sec, annealing at 60°C for 30 sec and extension at 72°C for 30 sec, followed by incubation at 72°C for 7 min. The amplified products were loaded onto a 1% agarose gel in Tris-acetate buffer for electrophoresis, stained with ethidium bromide, and then visualized under UV light.

### Immunoblotting

WT- and SD-Mg were plated on 100 mm dishes (1.5×10^6^ cells/dish) and then incubated overnight. The cells were washed twice with cold phosphate-buffered saline (PBS), and then 100 µL of lysis buffer [10 mM Tris-HCl (pH 7.5) containing 1% SDS, 1% Triton X-100, 1 mM NaF, 1 mM Na_3_VO_4_ and protease inhibitors (Complete protease inhibitor cocktail, Roche Diagnostics, Germany)] was added to them. The cells were harvested and then sonicated to prepare cell lysates. The protein concentration in each lysate was measured using a BCA protein assay kit (Pierce, Rockford, IL), and then an equal amount of protein was subjected to sodium dodecyl sulfate-polyacrylamide gel electrophoresis (SDS-PAGE) on a 10% acrylamide gel. Proteins were visualized by immunostaining with rabbit anti-signal transducer antibodies, HRP-labeled anti-mouse and rabbit IgG antibodies, and a chemiluminescence reagent (Immunobilon Western Reagent, Millipore, Bedford, MA). Immunoreactive bands on the blotts were quantified with a densitometer, LAS3000 (Fuji Film, Tokyo, Japan).

### Data analysis

Student's *t*-test was performed to evaluate the significance of the data. *P*<0.05 was considered statistically significant.

## Results

### PGE2 reduced MIP-1α production by SD-Mg

**Figure 1:**
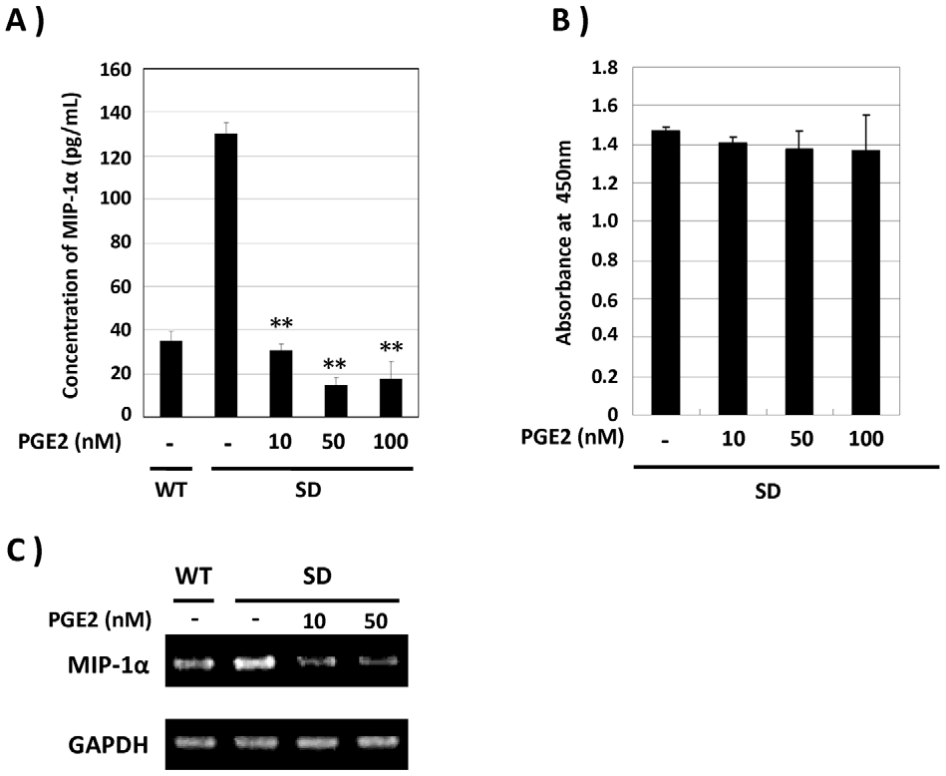
PGE2 reduces MIP-1α production by SD-Mg. **A**: SD-Mg was treated with the indicated concentrations of PGE2 for 6 h. The amounts of MIP-1α protein in CM were determined by ELISA. **B**: Cell viability was determined 6 h after treatment with PGE2. **C**: mRNA expression levels of MIP-1α in SD-Mg treated with PGE2 for 3 h were determined by RT-PCR. Values represent the means ± SD for three independent experiments. Significance was evaluated by means of Student's *t*-test. **P<0.01 versus controls (*t*-test).

In a previous study, we demonstrated that MIP-1α, a putative pathogenic factor for SD, was up-regulated in SD-Mg [11]. To determine whether or not PGE2 could inhibit the enhanced production of MIP-1α by SD-Mg, we first analyzed the effect of PGE2 on the production of MIP-1α by SD-Mg. The amount of MIP-1α in CM of SD-Mg on treatment with PGE2 was markedly decreased compared with of untreated SD-Mg (Fig. 1A). The reduction of the viability after treatment with the PGE2 was hardly observed (Fig. 1B). We next examined whether the inhibition of the enhanced MIP-1α production by PGE2 is under transcriptional control or not. As shown in Fig. 1C, PGE2 significantly reduced expression of the MIP-1α gene (Fig. 1C). These results indicate that PGE2 inhibits the production of MIP-1α by SD-Mg at both the transcriptional and translational levels.

### PGE2 attenuates activation of Akt and JNK involved in MIP-1α production by SD-Mg

**Figure 2:**
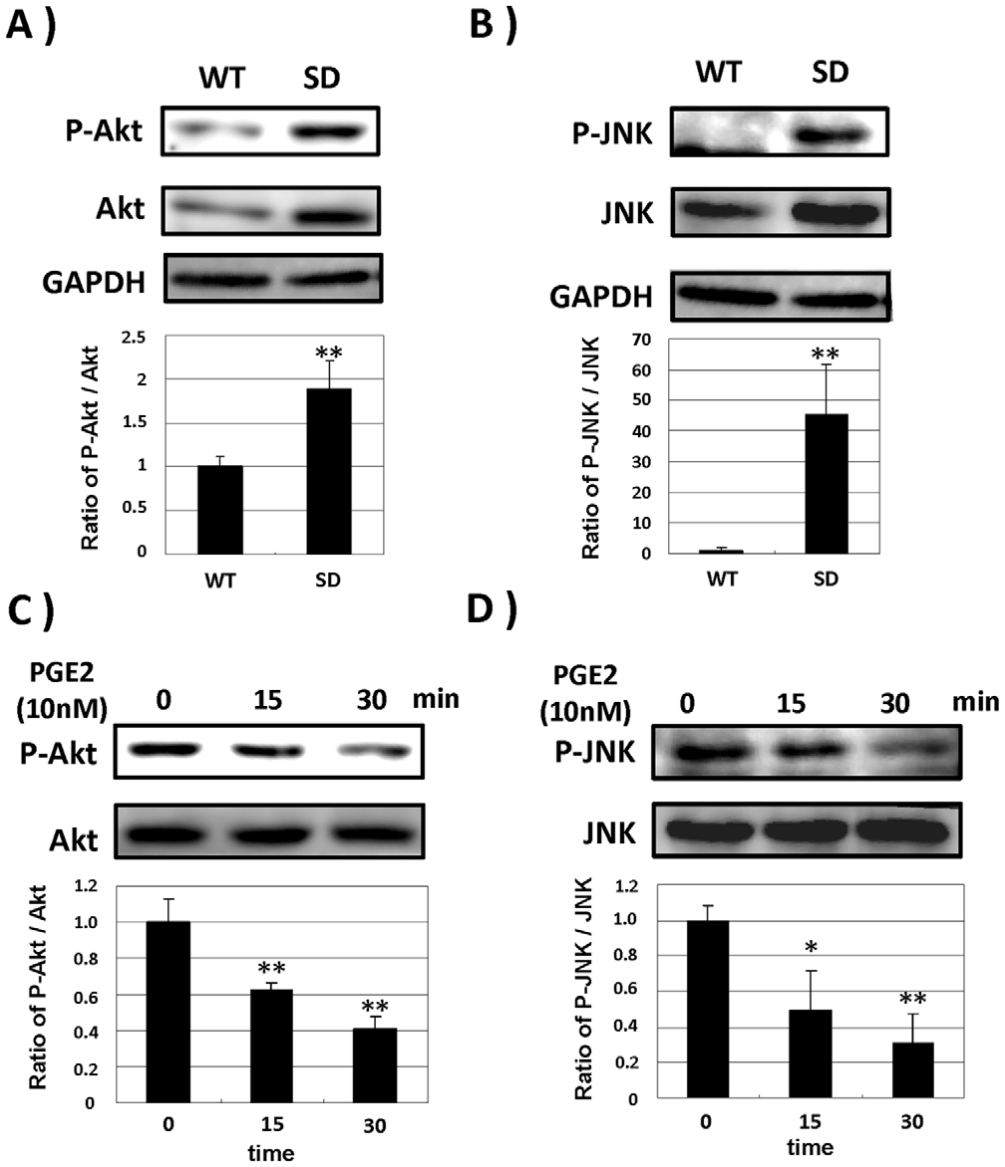
PGE2 attenuates activation of Akt and JNK in SD-Mg. **A**, **B**: Cell lysates were prepared with lysis buffer containing 1% SDS and 1% Triton X-100, and then subjected to immunoblotting using antibodies against Akt, JNK, phosphorylated Akt and JNK. **C**, **D**: SD-Mg was treated with 10 nM PGE2 for the indicated time periods. Phosphorylation of Akt and JNK was analyzed by immunoblotting. The histogram on the bottom panels represents the ratio of phorphorylated protein to total protein measured by densitometry. Values represent the means ± SD for three independent experiments. Significance was evaluated by means of Student's *t*-test. *P<0.05 and **P<0.01 versus WT-Mg or untreated SD-Mg.

We previously indicated that the enhanced activation of Akt and JNK mediated the enhanced production of MIP-1α [11]. To determine the mechanism underlying the reduction of MIP-1α in SD-Mg, the effects of PGE2 on the activation of Akt and JNK was analyzed. We defined the phosphorylation states of Akt and JNK in both WT- and SD-Mg by immunoblotting with anti-signaling molecules antibodies. The amounts of total and phosphorylated Akt protein were greater in SD-Mg than these in WT-Mg, and the ratio of phosphorylated Akt to total Akt in SD-Mg was significantly increased compared with that in WT-Mg (Fig. 2A). The total amounts of JNK protein in SD-Mg were also increased compared with that in WT-Mg (Fig. 2B). The phosphorylated JNK in WT-Mg was hardly detected, while that in SD-Mg was clearly detected. The ratio of phosphorylated JNK to total JNK in SD-Mg was significantly increased compared with that in WT-Mg (Fig. 2B). We next assessed the phosphorylation states of Akt and JNK in SD-Mg after treatment with PGE2 by immunoblotting. PGE2 significantly attenuated the phosphorylation of Akt and JNK in SD-Mg (Fig. 2C, D), suggesting that PGE2 reduces MIP-1α production by preventing the enhanced activation of Akt and JNK in SD-Mg.

### PGE2 reduced MIP-1α production by SD-Mg through binding with the EP2 and EP4 receptors

**Figure 3:**
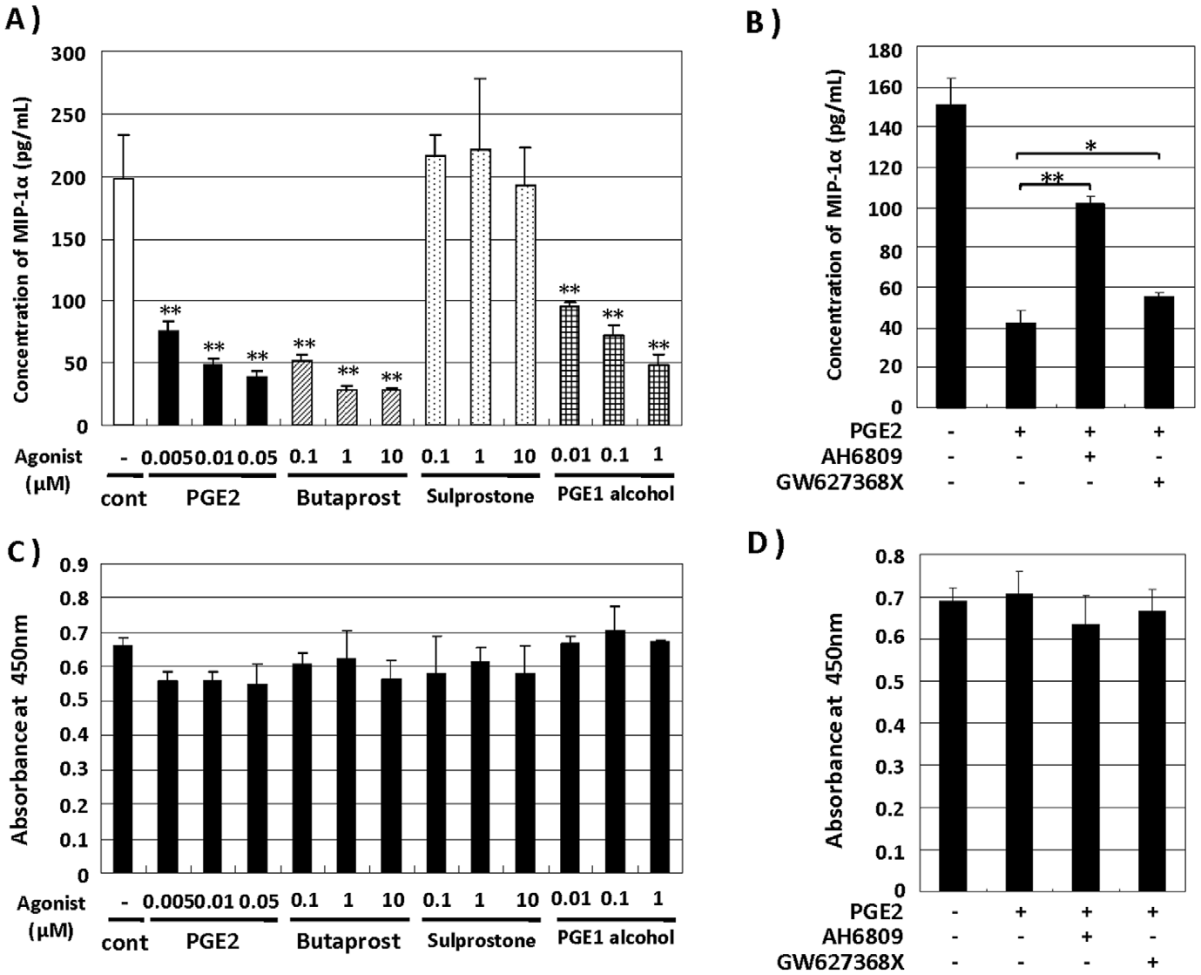
PGE2 reduces MIP-1α production by SD-Mg via putative EP2 and EP4 receptors. **A**: SD-Mg was treated with the indicated concentrations of PGE2, Butaprost, PGE1-alcohol and Sulprostone for 6 h. The amounts of MIP-1α protein in CM were determined by ELISA. **B**: SD-Mg was pretreated with the AH6809 and GW627368X at each 20 µM for 30 min, and then treated with 10 nM PGE2 for 6 h. The amounts of MIP-1α protein in CM were determined by ELISA. **C**, **D**: Cell viability was determined 6 h after treatment with the drugs. Values represent the means ± SD for three independent experiments. Significance was evaluated by means of Student's *t*-test. *P<0.05 and **P<0.01 versus controls.

PGE2 is known to possibly bind to four receptor subtypes (EP1, EP2, EP3 and EP4), inducing distinct signaling cascades [15]. To determine the detail mechanism underlying the reduction of MIP-1α in SD-Mg, we examined which PGE2 receptor subtypes contribute to the prevention of MIP-1α production by SD-Mg. We analyzed the effects of PGE2 analogs, Butaprost (EP2-selective agonist), PGE1 alcohol (EP3 and 4-selective agonist) and Sulprostone (EP1 and 3-selective agonist), on MIP-1α production by SD-Mg by means of ELISA. Butaprost and PGE1 alcohol significantly reduced the MIP-1α production, while Sulprostone had little effect on it (Fig. 3A). Reduction of the viability after treatment with all analogs was hardly observed (Fig. 3C). In addition, we analyzed the effects of EP2 and EP4-selective antagonists on the inhibition by PGE2 of MIP-1α production. AH6809 (EP2-selective antagonist) and GW627368X (EP4-selective antagonist) partially reversed the PGE2-induced suppression of MIP-1α production, and EP2-selective antagonist was more effective than EP4-selective one (Fig. 3B). All analogs had no effect on the viability (Fig. 3D). These results suggest that PGE2 reduces MIP-1α production by SD-Mg through binding with EP2 and EP4, but not EP1 and EP3.

### Activation of the adenylate cyclase pathway was involved in the reduction of MIP-1α production by SD-Mg

**Figure 4:**
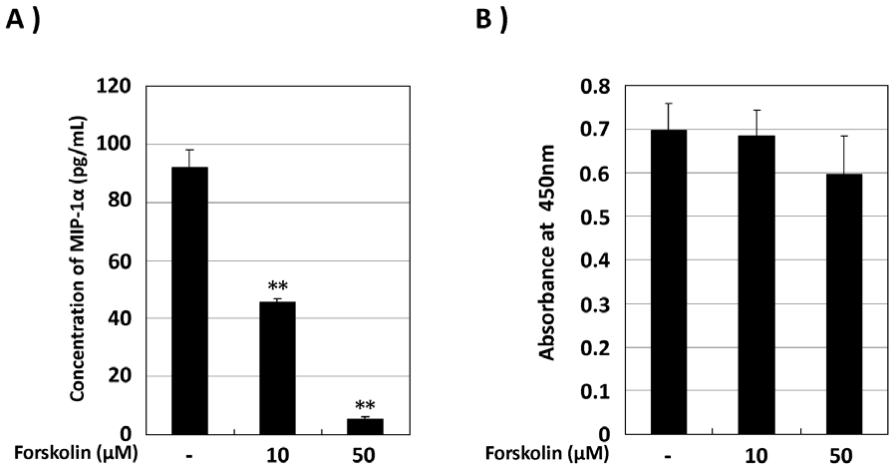
Activation of adenylate cyclase inhibits MIP-1α production by SD-Mg. **A**: SD-Mg was treated with forskolin, an adenylate cyclase activator, for 6 h at the indicated concentrations. The amounts of MIP-1α protein in CM were determined by ELISA. **B**: Cell viability was determined 6 h after treatment with forskolin. Values represent the means ± SD obtained for three independent experiments. **P<0.01 versus untreated control (*t*-test).

We demonstrated that PGE2 inhibited the induction of MIP-1α in SD-Mg through binding with EP2 and EP4. As EP2 and EP4 couple to Gα_s_ to activate adenylate cyclase (AC) [15], we examined whether forskolin, an AC activator, mediates the reduction of MIP-1α production by SD-Mg or not. The level of MIP-1α secretion by SD-Mg was decreased on activation of AC in a dose-dependent manner (Fig. 4A). Forskolin had little effect on cell viability (Fig. 4B). These data suggest that the increase in intracellular cAMP caused by the activation of AC is involved in the induction of MIP-1α production by SD-Mg.

### cAMP/PKA reduced MIP-1α production by attenuating of Akt and JNK activation in SD-Mg

**Figure 5:**
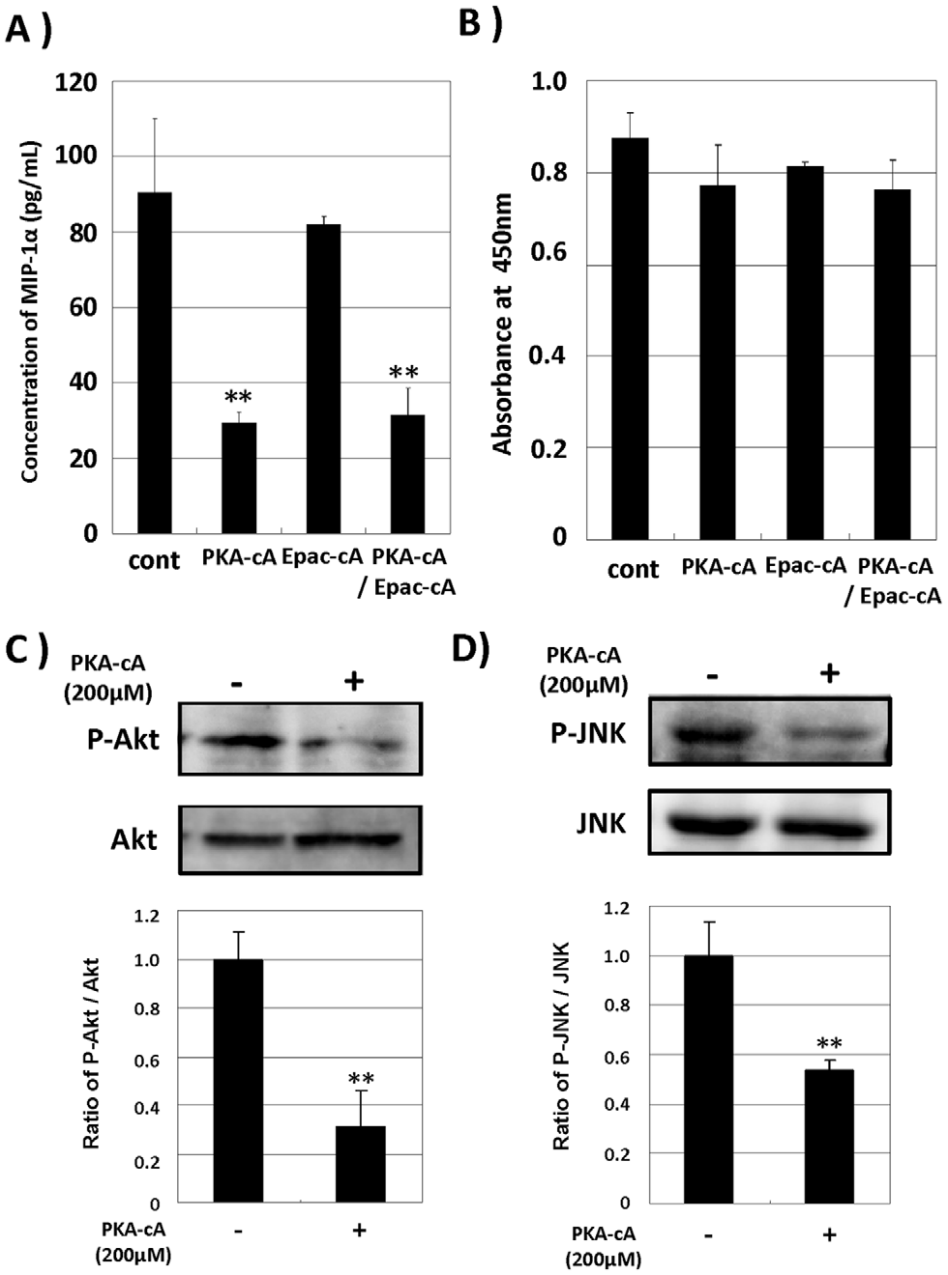
Activation of cAMP/PKA reduces MIP-1α production by SD-Mg through dephosphorylation. **A**: SD-Mg was treated with cAMP analogs, the 6-Bnz-cAMP (PKA-selective; PKA-cA) and 8-pCPT-2′O-Me-cAMP (Epac-selective; Epac-cA) analogs, at 200 µM alone or in combination for 6 h. The amounts of MIP-1α protein in CM were determined by ELISA. Values represent the means ± SD for three independent experiments. **P<0.01 versus untreated controls (*t*-test). **B**: Cell viability was determined 6 h after treatment with the cAMP analogs. **C**, **D**: SD-Mg was treated with 200 µM PKA-selective cAMP for 30 min. Phosphorylation states of Akt and JNK were analyzed by immunoblotting. The histogram on the bottom panels represents the ratio of phorphorylated protein to total protein measured by densitometry. Values represent the means ± SD for three independent experiments. Significance was evaluated by means of Student's *t*-test. **P<0.01 versus WT-Mg.

The primary transducer of the cellular response to cAMP is cAMP-dependent protein kinase (PKA), but exchange proteins directly activated by cAMP (Epac) has been recently discovered as a signaling protein that bind cAMP and activate the small Ras-related monomeric G proteins Rap1 and Rap2 [23], [24]. To assess the involvement of PKA or Epac in the reduction of MIP-1α production, we utilized newly developed cAMP analogs selective for PKA and Epac, respectively [25], [26]. The PKA-selective cAMP analog significantly reduced MIP-1α production by SD-Mg, while the Epac-selective one had no effect. In addition, when SD-Mg was treated with the PKA- and Epac-selective analogs in combination, no additive effect was observed on reduction of MIP-1α production (Fig. 5A). Cell viability was confirmed to be well maintained on treatment with the cAMP analogs (Fig. 5B). These results indicate that the cAMP/PKA pathway, but not the cAMP/Epac one, mediates the reduction of MIP-1α production. As shown in Fig. 2, PGE2 reduced MIP-1α production through attenuation of Akt and JNK signaling in SD-Mg. It is likely that the attenuation by PGE2 may be involved in cAMP/PKA activation. We next assessed the phosphorylation states of Akt and JNK in SD-Mg after treatment with the PKA-selective cAMP analog by immunoblotting. The activation of Akt and JNK was attenuated by treatment with the PKA-selective cAMP analog (Fig. 5C, D). These results suggest that activation of the cAMP/PKA pathway reduced MIP-1α production through attenuation of Akt and JNK signaling in SD-Mg.

## Discussion

Microglia-mediated inflammation in the CNS has been observed in the brains of SD patients and model mice, and contributes to the pathogenesis and progression of SD [6]–[9]. Our previous studies demonstrated that the production of MIP-1α is enhanced in microglia of SD mice *in vivo* and *in vitro* [10], [11]. It has also been reported that an increase in life expectancy was observed in Hexb^−/−^ MIP-1α^−/−^ double knockout mice compared with in Hexb^−/−^ mice [12]. These studies suggest that MIP-1α is a critical factor in the neuropathogenesis of SD, and that it is important to prevent MIP-1α production by microglia in order to delay the onset or progression of SD. However, no study on the down-regulation of the abnormal production of MIP-1α in SD-Mg has yet been examined. PGE2 has been shown to reduce the production of MIP-1α by LPS-stimulated immune cells, including macrophage and dendritic cells [21], [22]. In this study, we therefore investigated the inhibitory effect of PGE2 on the abnormal production of MIP-1α in SD-Mg. PGE2 reduced MIP-1α productionby SD-Mg to the same level as by WT-Mg (Fig. 1), suggesting that PGE2 plays a role as a negative regulator of MIP-1α production by SD-Mg. Our previous study demonstrated that the activation of Akt and JNK in SD-Mg mediates the abnormal production of MIP-1α, as evidenced by a decrease in MIP-1α production by treatment with the pharmacological inhibitors [11]. In fact, this current study demonstrated that the activation of Akt and JNK was enhanced in SD-Mg compared with in WT (Fig. 2A). Interestingly, PGE2 significantly attenuated the enhanced activation of Akt and JNK in SD-Mg (Fig. 2B), suggesting that PGE2 exerts the inhibitory effect on the MIP-1α production by normalizing the phosphorylation state of Akt and JNK. Therefore, the findings suggest that PGE2-mediated signaling pathway is the potential therapeutic target for reducing the MIP-1α production in the brain of SD.

PGE2 is widely known as an inflammatory mediator, while it could also have anti-inflammatory properties [14]. The physiological functions of PGE2 depend on the receptor subtype, EP1-4, each coupling to the opposing second messenger [15]. It is important to understand the detail mechanism underlying the inhibition by PGE2 of MIP-1α production by microglia in order to prevent an adverse effect in developing the treatment for SD patients, which has not been studied. We found in this study that EP2 and EP4, but not EP1 and EP3, could be involved in the inhibitory effect of PGE2 on MIP-1α production by SD-Mg (Fig. 3A). The EP2-selective antagonist was more effective in reversing the production than EP4-selective one (Fig. 3C), suggesting that EP2 may be responsible for the inhibition by PGE2. We also demonstrated that reduction of the MIP-1α production by PGE2 was mediated through the cAMP/PKA pathway, but not the cAMP/Epac one (Fig. 5A). In addition, it was shown that the activation of the cAMP/PKA pathway could attenuate the enhanced activation of Akt and JNK in SD-Mg (Fig. 5C, D). Taken together, PGE2 can suppress MIP-1α production by preventing the activation of Akt and JNK through the EP2 and 4/cAMP/PKA pathway in SD-Mg. In dendritic cell, PGE2 was reported to inhibit LPS-induced MIP-1α production by the activation of Akt through the cAMP/Epac pathway, but not the cAMP/PKA one [27]. It is interesting that the activation of PKA and Epac can inhibit the production of MIP-1α, although they have opposite effects on regulation of the activation of Akt. Difference in their inhibitory effects might be due to the phosphorylation state of Akt within cells. Although we did not elucidate the reason why PKA rather than Epac is involved in the inhibition in microglia, this can be explored in future studies. However, we first elucidated the mechanism underlying the inhibition by PGE2 of MIP-1α production in microglia.

Therapeutic approaches for SD have been investigated, including substrate reduction therapy [28], [29], bone marrow transplantation [30], [31], stem cell therapy [32] and enzyme replacement therapy [33], in which the aim is to reduce the accumulated substrates. The treatment may be more effective to delay the progression of SD by a combination therapy with the reduction of MIP-1α production. In this study, we first demonstrated that the activation of EP2 and EP4/cAMP/PKA pathway suppresses the abnormal production of MIP-1α in SD-Mg. Although further research is necessary, our findings may lead to the future therapeutic application for SD.
